# Relative biological effectiveness of the 60-MeV therapeutic proton beam at the Institute of Nuclear Physics (IFJ PAN) in Kraków, Poland

**DOI:** 10.1007/s00411-014-0559-0

**Published:** 2014-07-19

**Authors:** Dorota Słonina, Beata Biesaga, Jan Swakoń, Damian Kabat, Leszek Grzanka, Marta Ptaszkiewicz, Urszula Sowa

**Affiliations:** 1 Department of Applied Radiobiology, Centre of Oncology, Maria Skłodowska-Curie Memorial Institute, Garncarska 11, 31-115 Kraków, Poland; 2 Institute of Nuclear Physics, Polish Academy of Sciences, Kraków, Poland; 3 Department of Medical Physics, Centre of Oncology, Maria Skłodowska-Curie Memorial Institute, Kraków, Poland

**Keywords:** Proton beam, Spread-out Bragg peak, RBE, Human fibroblasts

## Abstract

The aim of the study was to determine the relative biological effectiveness (RBE) of a 60-MeV proton radiotherapy beam at the Institute of Nuclear Physics, Polish Academy of Sciences (IFJ PAN) in Kraków, the first one to operate in Poland. RBE was assessed at the surviving fractions (SFs) of 0.01, 0.1, and 0.37, for normal human fibroblasts from three cancer patients. The cells were irradiated near the Bragg peak of the pristine beam and at three depths within a 28.4-mm spread-out Bragg peak (SOBP). Reference radiation was provided by 6-MV X-rays. The mean RBE value at SF = 0.01 for fibroblasts irradiated near the Bragg peak of pristine beam ranged between 1.06 and 1.15. The mean RBE values at SF = 0.01 for these cells exposed at depths of 2, 15, and 27 mm of the SOBP ranged between 0.95–1.00, 0.97–1.02, and 1.05–1.11, respectively. A trend was observed for RBE values to increase with survival level and with depth in the SOBP: at SF = 0.37 and at the depth of 27 mm, RBE values attained their maximum (1.19–1.24). The RBE values estimated at SF = 0.01 using normal human fibroblasts for the 60-MeV proton radiotherapy beam at the IFJ PAN in Kraków are close to values of 1.0 and 1.1, used in clinical practice.

## Introduction

To date, some 94,000 patients have received proton therapy in 36 proton therapy centres worldwide (Particle Therapy Co-Operative Group 2012). In 2011, a 60-MeV proton radiotherapy facility, the first one to operate in Poland, began to treat patients with eye tumours (mainly uveal melanoma) at the Institute of Nuclear Physics of the Polish Academy of Sciences (IFJ PAN) in Kraków, Poland. The proton beam is accelerated using the AIC-144 isochronous cyclotron, which was designed and constructed around 1980 at the IFJ PAN for physics research (Bakewicz et al. 2003) and adapted around 2008 to supply a 60-MeV proton beam to the proton ocular treatment facility (Swakon et al. 2010). The first patient was irradiated at IFJ PAN in February 2011 and by June 2014, 62 patients were treated.

The physical advantage of protons is the spatial distribution of dose delivered to the tumour volume. Protons and heavier ions show an increasing energy loss with depth in tissue that they penetrate, leading to maximum dose deposition, known as the Bragg peak, at the distal range of the proton beam. For protons, no significant dose is deposited at depths exceeding that of the Bragg peak. Such physical properties of ions are desirable in radiotherapy because they allow targeting the therapeutic dose within the tumour volume with high accuracy, sparing normal tissues and critical organs located beyond the beam’s range. Therefore, patients with tumours located close to critical organs are preferentially referred to proton centres. For the purposes of radiotherapy, the Bragg peak in a pristine beam is too narrow to homogeneously cover the tumour volume; therefore, beam energy modulation is required to produce a spread-out Bragg peak (SOBP; Paganetti and Bortfeld 2006).

The radiobiological properties of a therapeutic proton beam are comparable to those of a photon beam. A generic value of relative biological effectiveness (RBE) of 1.1 is accepted and usually applied in routine proton radiotherapy, independently of the initial beam energy, position within the SOBP, dose fractionation scheme, or type of irradiated tissue (ICRU 78 2007). Although it has been recognised that these physical and biological factors affect the RBE value (Gerweck and Kozin 1999; Paganetti and Goitein 2000; Paganetti et al. 2002), they are usually disregarded in clinical proton therapy practice. Most authors show that RBE increases with depth along the SOBP, due to the increase in the linear energy transfer (LET). For human tumour cells in vitro, RBE values reach 1.2–1.3 at the distal part of the SOBP (Courdi et al. 1994; Bettega et al. 2000; Ando et al. 2001; Calugaru et al. 2011), although recently Britten et al. (2013) found a much higher value of 2.1. Due to this increase, the biological effective range of the proton beam may extend by about 1–3 mm over its physical range (Bettega et al. 2000; Paganetti and Goitein 2000; Carabe et al. 2012). Thus, critical organs located just behind the target volume may be affected. It was suggested that this extension of the effective beam range depends on dose and tissue type (i.e. on its *α*/*β* value), and that it should be considered for low doses and tissues characterised by low *α*/*β* ratios (Gerweck and Kozin 1999). The clinical relevance of the RBE variation and range uncertainty of the proton beam is still a matter of debate (Carabe et al. 2012; Dasu and Toma-Dasu 2013; Frese et al. 2011; Paganetti 2012).

It was shown that beam-modulating devices for proton therapy may affect the dose distribution and the proton energy spectrum of the beam, and consequently its RBE (Paganetti and Goitein 2000). Because different therapy centres apply different beam modulation systems, prior to their clinical use, independent radiobiological assessment of the RBE of each proton beam at each centre is required.

To date, normal human cells in vitro have not been extensively used for determining the RBE values for proton radiotherapy beams. Here, we report the results of radiobiological studies performed in vitro using primary normal fibroblasts derived from three patients with cervix cancer. Our aim was to evaluate the RBE value for the IFJ PAN 60-MeV proton beam and its dependence on the level of survival (dose level) and position in the SOBP or within the Bragg peak of the pristine beam. Skin fibroblasts were chosen firstly because they represent target cells of late-responding tissue (fibrosis) and thus exhibit a low *α*/*β* ratio (Bentzen and Joiner 2009), and secondly, because of their known high individual variation in radiosensitivity (Peacock et al. 2000; Słonina et al. 2007).

## Materials and methods

### Proton beam and dosimetry

Cell irradiation was performed at the IFJ PAN proton beam facility used for the therapy of uveal melanoma. The 60-MeV proton beam is accelerated in the AIC-144 isochronous cyclotron (Bakewicz et al. 2003). Protons are transported by a 25-m-long beam line system to the radiotherapy room. The beam line system consists of evacuated pipes of 10-cm internal diameter, bending magnets, and a system of quadrupole and correction magnets. The horizontal proton beam is spread out laterally using a single scattering system with a 25-μm tantalum foil located 10 m upstream of the isocentre of the therapy facility. The proton beam forming system is located on an optical bench placed in the treatment room. The beam is formed using a poly-methyl methacrylate (PMMA—water-equivalent material) range shifter, and a rotating range modulator, which forms a flat SOBP. A six-sector ionisation chamber operating with a dedicated set of electrometers controls the beam homogeneity and stability, while two PTW TM7862 monitor ionisation chambers (PTW, Freiburg, Germany) connected to electrometers verify the dose. The main specifications of the IFJ PAN proton radiotherapy beam are dose rate—variable over the range 0.25–1.0 Gy/s; range in water—28.4 mm (defined as 90 % of the maximum at the distal edge) and distal fall-off—0.75 mm (defined as the distance between 90 and 10 % of the maximum). The diameter of the irradiation field is up to 40 mm. Details of the facility and of its beam geometry and physical characteristics have been reported elsewhere (Swakon et al. 2010).

**Fig. 1:**
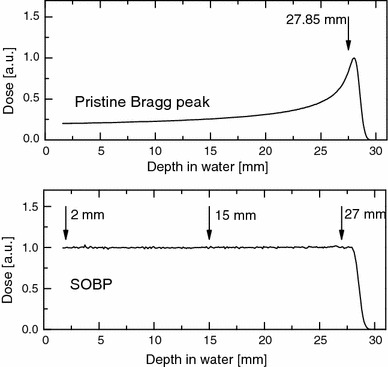
Depth–dose distributions of the proton beam produced at the IFJ PAN Kraków (nominal energy 60 MeV) measured in a water phantom using a Marcus ion chamber. *Upper panel*: pristine beam; *lower panel*: SOBP. *Arrows* indicate positions at which cells (human fibroblasts) were irradiated: near (at 27.85 mm) the maximum of the Bragg peak in a pristine beam, and at three depths (2, 15, and 27 mm) of the SOBP of full width 28.4 mm

In the experiments reported here, a pristine proton beam with a Bragg peak maximum at 28.4 mm depth in water and 0.75 mm distal fall-off, and a modulated Bragg peak (SOBP) over the full beam range, were applied. A thirty-six gradual, four-sectored rotating PMMA propeller was used to produce the full-range SOBP. Cells were irradiated near the pristine beam’s Bragg peak (at 27.85 mm depth) and at three depths (2, 15, and 27 mm) of the 28.4-mm SOBP. Figure 1 shows the depth–dose distributions measured in a water phantom for the pristine beam and for the SOBP, indicating the irradiation positions. All the experiments were performed with a 40-mm-diameter collimated beam of circular cross section. Beam dosimetry was performed according to the International Atomic Energy Agency (IAEA) protocol TRS-398 (2000), using a standard kit consisting of a PTW TM23343 Markus chamber and a PTW UNIDOS electrometer (PTW, Freiburg, Germany) both calibrated by a ^60^Co source at the IFJ PAN.

### Calculation of dose-averaged linear energy transfer (LETd)

**Fig. 2:**
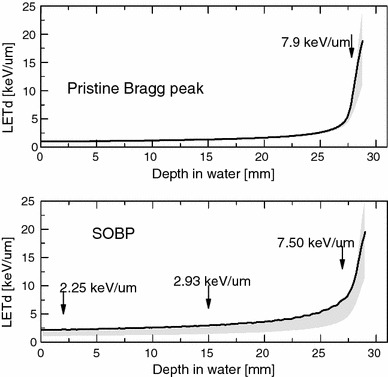
Depth distributions of dose-averaged LET (LET_d_) and of IQR of LET_d_ (*shaded*) of the proton beam produced at the IFJ PAN, simulated by Monte Carlo calculations. *Upper panel*: pristine beam; *lower panel*: SOBP. Calculated values of LET_d_ at irradiation positions are also shown

Numerical simulations of the IFJ PAN proton beam were performed using the Geant 4 9.6 Monte Carlo code, optimised for medical physics applications by ‘EM opt3’ settings (Agostinelli et al. 2003). A pristine Bragg peak of 28.4 mm range and fully SOBP corresponding to that measured were simulated. Simulations of the proton beam were performed in liquid water. Spread-out Bragg peak simulations included the correction factors based on the measurements of the proton beam passing through PMMA range modulator and optical bench. Among the quantities scored as a function of depth were absorbed dose, the energy spectrum, and the LET spectrum. As a measure of the central tendency of the LET spectrum, the weighted mean was taken, i.e. the dose-averaged LET (LET_d_). This spectrum was found to have a skewed distribution; thus, the interquartile range (IQR) was taken as a measure of its spread. Figure 2 shows the depth distributions of LET_d_ and of their IQR for the pristine Bragg peak and for the SOBP. The respective values of LET_d_ at irradiation positions are also shown. The calculated value of LET_d_ near the pristine Bragg peak maximum at 27.85 mm depth (90 % of the maximum at the proximal edge) was 7.9 keV/µm (equivalent to a proton energy of 5.7 MeV) with IQR = 3.26 keV/µm. The LET_d_ values calculated at three depths 2, 15, and 27 mm of the SOBP were 2.25 keV/µm (38.0 MeV) with IQR = 1.1; 2.93 keV/µm (26.3 MeV) with IQR = 1.78; and 7.50 keV/µm (8.6 MeV) with IQR = 4.19 keV/µm, respectively, as shown in Fig. 2.

### Cell cultures

Primary human fibroblasts from three patients with cervix cancer (HFIB2, HFIB15, HFIB30) were irradiated. The cells originated from normal skin fragments are taken from the pelvic area of the patients during hysterectomy. Informed consent was obtained from the patients. The current study was approved by the Ethical Committee of the Regional Medical Chamber in Kraków. The procedure for obtaining and preparation of the cells was described previously (Słonina et al. 2007, 2008). We used fourth- to sixth-passage fibroblasts. Fibroblasts were grown as a monolayer in 75-cm^2^ culture flasks (Nunc, Roskilde, Denmark) containing Dulbecco’s modified eagle medium (DMEM) supplemented with 10 % foetal calf serum (FCS), 1 % hydroxyethylpiperazine ethanesilfonate (HEPES), 1 % sodium pyruvate (all Biochrom, Berlin, Germany), and antibiotics (100 U/ml penicillin, 100 μg/ml streptomycin, and 0.25 μg/ml amphotericin B; Sigma, Roskilde, Denmark). The cells were incubated at 37 °C in a humidified atmosphere of 95 % air/5 % CO_2_ and subcultured before they reached 70 % confluence.

Two days before irradiation, two sets of cells in exponential growth were seeded for each experiment (one for proton and one for reference X-ray exposures), two dishes per dose point. For proton irradiation, cells were plated in a drop of medium (at a density of 10^5^ cells per 400 µl) centrally on 35-mm Petri dishes (Nunc, Roskilde, Denmark) and left overnight to attach within a small area (~1 cm^2^). Thereafter, the culture dishes were filled with 2 ml medium. Such a procedure resulted in dose homogeneity over the cell area to within ±3 %. For X-ray irradiation, cells were seeded (at a density of 10^5^ cells/ml) onto 35-mm Petri dishes with 2 ml of medium. On the day of irradiation, one set of cells was transported to the IFJ PAN for proton exposure, while the second set was irradiated at the same time with X-rays at the Centre of Oncology. All irradiations and transports of cells were carried out at room temperature (22 ± 2 °C), and all experiments concerning fibroblasts of each patient were repeated three to five times.

### Proton irradiation

For proton irradiation, a specially designed PMMA block (15-mm thick) containing the space for one 35-mm Petri dish was used. The proton beam at the IFJ PAN is a horizontal one; therefore, to face the beam, the Petri dish with cells was placed in the PMMA block in vertical position. The surface with cells was placed at the isocentre of the proton beam at a distance of 93 mm from the final collimator. The culture medium was discarded just before and added just after irradiation, so the cells stayed without medium over periods no longer than 5 min. We verified, by comparison with cells irradiated with the medium present, that this procedure had no effect on cell survival. Cells were positioned at the specified depths of the proton beam by placing a PMMA plate of appropriate thickness in front of the Petri dish. The water equivalent for each PMMA plate was previously estimated in proton beam. The precision of cell positioning was better than ±0.1 mm. The cells were irradiated at each position with single exposures with doses ranging from 0 to 8 Gy, at a mean dose rate of 12 Gy/min. To control the proton beam parameters during irradiation, two PTW 7862 ionisation chambers and a six-sector ionisation chamber connected to a dedicated set of electrometers were used.

### X-ray irradiation

A 6-MV X-ray beam from a linear accelerator (Varian Clinac 600CD) was used for reference irradiation. For X-ray irradiation, Petri dishes with cells were placed in a special PMMA block (30 × 30 cm) fitted to hold four 35-mm dishes. The dose was delivered by a 15 × 15 cm^2^ collimated field at source to surface (SSD) of 100 cm. The photon beam was directed upwards to irradiate the Petri dishes from below. Dose homogeneity was maintained within ±1.5 %. Doses ranging from 0 to 8 Gy were delivered at a dose rate of 3 Gy/min. Dosimetry was performed using a PPC05 parallel plate ionisation chamber and a DOSE-1 electrometer. The photon beam was calibrated using the IAEA protocol TRS-398 (2000).

### Clonogenic survival assay

The cells irradiated with protons and X-rays were processed at the same time. About 1 h after irradiations, the cells were detached with trypsin/ethylenediaminetetraacetic acid (EDTA) (0.05 %/0.02 %, Biochrom, Berlin, Germany) and counted. Depending on the irradiation dose and the expected SF, an appropriate number of cells (500–50,000) was plated onto 100-mm Petri dishes (Falcon; Franklin Lakes, USA, three dishes per dose) in 15 ml of medium, to give 50–100 colonies per dish. After incubation at 37 °C for 18 days, the resultant colonies were washed with 0.9 % sodium chloride (NaCl), fixed in 80 % ethanol (both Chempur, Piekary Śląskie, Poland) and stained with 1 % crystal violet (Merck, Darmstadt, Germany). Colonies consisting of more than 50 cells were scored as representing surviving cells. The SF at each dose point was calculated as an average of three plates. The plating efficiency (PE) of non-irradiated HFIB2 cells was ~25 %, while that of HFIB15 and HFIB30 was ~30 and ~21 %, respectively. SF was calculated as the ratio of PE for irradiated and non-irradiated cells.

### Analysis of cell survival data and RBE calculation

The clonogenic survival data were fitted to the linear-quadratic formula (LQ):

$${\text{SF}} = \exp ( - \alpha d - \beta d^{2} )$$

where *d* is the physical dose. Multiple regression analysis was used to calculate the *α* and *β* values for each independent experiment (Statistica 10.0). Doses required to reduce the SF to 0.01, 0.1 or 0.37 were computed using these *α* and *β* values. Then, the RBE values were obtained by comparing the doses of 6-MV X-rays with those of protons at each survival level. Finally, the mean values of *α* and *β* and their standard deviations (SDs), as well as respective RBE values were calculated from 3 to 5 independent experiments using the fibroblasts of each patient. The dependence of RBE on survival level, depth in the SOBP, and individual patient was tested by analysis of variance. A *p* value of <0.05 was considered to be statistically significant.

## Results

### Cell survival near the Bragg peak maximum

**Fig. 3:**
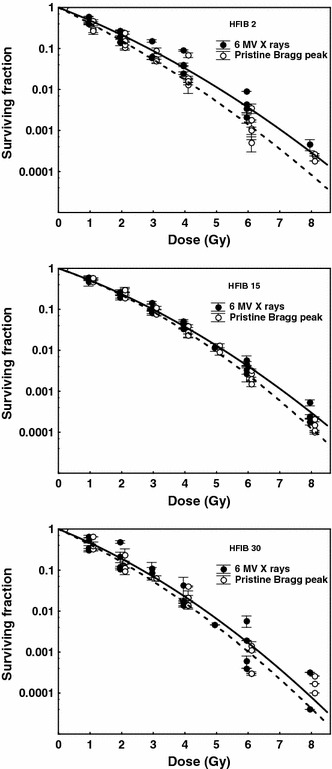
Cell survival curves for fibroblasts of three patients (HFIB2, HFIB15, and HFIB30) after irradiation with 6-MV X-rays (*solid line*) and by 60 MeV protons near the maximum of the Bragg peak in a pristine beam (*dashed line*). Each point represents the mean value ± SD from one experiment (three culture dishes)

The clonogenic cell survival curves for fibroblasts of three patients HFIB2, HFIB15, and HFIB30 irradiated near the maximum of the Bragg peak are shown in Fig. 3 and compared with the reference curves for X-rays. For fibroblasts of each patient, lower survival after proton irradiation compared with that after X-rays was observed. Table 1 contains values of α and β parameters derived from the LQ formula, the SFs at 2 Gy (SF2), and RBE values calculated at three survival levels, SF = 0.01, 0.1, or 0.37, corresponding to the doses of about 5, 3, and 1 Gy, respectively. The mean RBE values for HFIB2, HFIB15, and HFIB30, at the position near the Bragg peak maximum, exceeded 1.0. At SF = 0.01, RBE values of 1.15, 1.06, and 1.11, respectively, were found. An increase in the mean RBE value with increasing survival level from 0.01 to 0.37 (or with decreasing dose from about 5 to about 1 Gy), although seen for fibroblasts of two patients (up to 1.21 for HFIB2 and 1.16 for HFIB30), was not significant (*p* = 0.3918 and 0.9166, respectively). The differences in RBE values between cells from the three patients were not significant at the studied SF levels of SF = 0.01 (*p* = 0.7952), 0.1 (*p* = 0.4990) or SF = 0.37 (*p* = 0.3024).

**Table 1:** Survival parameters and RBE values for normal human fibroblasts irradiated in a pristine 60-MeV proton beam near its Bragg peak

Irradiation	*α* (Gy^−1^)	*β* (Gy^−2^)	SF2	RBE SF = 0.37	RBE SF = 0.1	RBE SF = 0.01
HFIB 2						
6-MV X-rays	0.689 ± 0.164	0.041 ± 0.015	0.21 ± 0.05			
Pristine Bragg peak	0.855 ± 0.252	0.040 ± 0.047	0.15 ± 0.05	1.21 ± 0.05	1.19 ± 0.05	1.15 ± 0.09
HFIB 15						
6-MV X-rays	0.627 ± 0.028	0.048 ± 0.014	0.22 ± 0.04			
Pristine Bragg peak	0.630 ± 0.033	0.062 ± 0.005	0.25 ± 0.05	1.03 ± 0.04	1.05 ± 0.03	1.06 ± 0.04
HFIB 30						
6-MV X-rays	0.718 ± 0.217	0.058 ± 0.017	0.24 ± 0.17			
Pristine Bragg peak	0.814 ± 0.141	0.056 ± 0.041	0.16 ± 0.08	1.16 ± 0.29	1.14 ± 0.30	1.11 ± 0.33

Listed are mean values ± SD from 4 to 5 independent experiments

### Cell survival at three depths of the SOBP

**Fig. 4:**
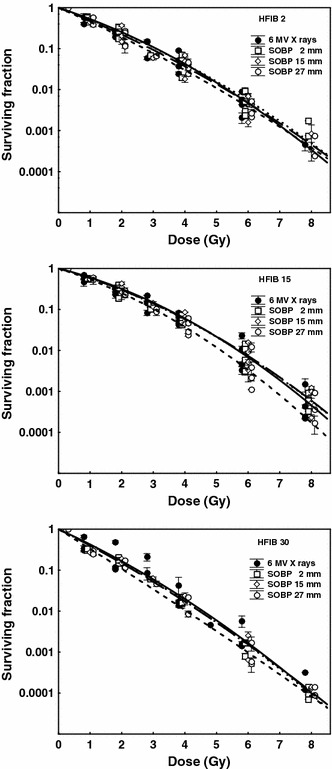
Cell survival curves for fibroblasts of three patients (HFIB2, HFIB15, and HFIB30) after irradiation with 6-MV X-rays (*solid line*) and by 60 MeV protons at three depths of the SOBP: 2 mm (*dotted line*), 15 mm (*long-dashed line*), and 27 mm (*dashed line*). Each point represents the mean value ± SD from one experiment (three culture dishes)

Figure 4 depicts the survival curves for fibroblasts HFIB2, HFIB15, and HFIB30 irradiated at three different depths (2, 15, and 27 mm) of the 60-MeV SOBP and with X-rays. For fibroblasts of each patient, the survival curves after proton irradiation at 2 and 15 mm of the SOBP and for X-rays were almost identical, while protons at 27 mm depth killed fibroblasts slightly more efficiently than X-rays. Parameters obtained from fitting cell survival data with the LQ formula and RBE values for protons relative to X-rays derived from these curves and calculated at three survival levels are presented in Table 2. The mean RBE values calculated at SF = 0.01 (~5 Gy) lied between 0.95 and 1.02 up to 15 mm in depth and increased at the depth of 27 mm to 1.06, 1.11, and 1.05 for HFIB2, HFIB15, and HFIB30, respectively. For each patient at any studied survival level, the RBE values increased at the distal part (27 mm) of the SOBP. The highest enhancement was observed at SF = 0.37 attaining mean RBE values of 1.23, 1.19, and 1.24 for HFIB2, HFIB15, and HFIB30, respectively. However, only in the case of HFIB30, RBE at depth of 27 mm was significantly higher than that at 2 mm (*p* = 0.0328, 0.0412, 0.0393 at SF 0.37, 0.1, and 0.01, respectively). The differences between RBE measured at depths of 2 and 15 mm, and at 15 and 27 mm, were not significant. Regarding the RBE–dose relationship, at each depth of the SOBP, the RBE value gradually increased with survival level; however, the growth was significant only for HFIB30 cells irradiated at the depth of 27 mm (*p* = 0.0370). For HFIB30 cells, the mean RBE value increased from 1.05 (at SF = 0.01) to 1.24 (at SF = 0.37).

**Table 2:** Survival parameters and RBE values for normal human fibroblasts irradiated at three depths in a 60-MeV SOBP

Irradiation	*α* (Gy^−1^)	*β* (Gy^−2^)	SF2	RBE SF = 0.37	RBE SF = 0.1	RBE SF = 0.01
HFIB 2						
6-MV X-rays	0.582 ± 0.148	0.052 ± 0.008	0.24 ± 0.03			
SOBP 2 mm	0.620 ± 0.159	0.041 ± 0.018	0.26 ± 0.07	1.04 ± 0.10	1.01 ± 0.07	0.98 ± 0.06
SOBP 15 mm	0.686 ± 0.273	0.035 ± 0.029	0.24 ± 0.10	1.11 ± 0.13	1.07 ± 0.08	1.02 ± 0.03
SOBP 27 mm	0.772 ± 0.177	0.026 ± 0.020	0.19 ± 0.08	1.23 ± 0.22	1.15 ± 0.15	1.06 ± 0.08
HFIB 15						
6-MV X-rays	0.446 ± 0.129	0.065 ± 0.010	0.28 ± 0.06			
SOBP 2 mm	0.500 ± 0.014	0.054 ± 0.018	0.31 ± 0.09	1.07 ± 0.21	1.04 ± 0.17	1.00 ± 0.15
SOBP 15 mm	0.493 ± 0.098	0.054 ± 0.021	0.32 ± 0.09	1.06 ± 0.11	1.02 ± 0.11	1.00 ± 0.13
SOBP 27 mm	0.561 ± 0.100	0.065 ± 0.032	0.25 ± 0.04	1.19 ± 0.14	1.14 ± 0.17	1.11 ± 0.10
HFIB 30						
6-MV X-rays	0.814 ± 0.125	0.042 ± 0.018	0.25 ± 0.06			
SOBP 2 mm	0.861 ± 0.112	0.038 ± 0.016	0.18 ± 0.03	1.02 ± 0.10	0.99 ± 0.07	0.95 ± 0.05
SOBP 15 mm	0.894 ± 0.125	0.031 ± 0.006	0.17 ± 0.02	1.05 ± 0.05	1.01 ± 0.02	0.97 ± 0.00
SOBP 27 mm	1.096 ± 0.141	0.010 ± 0.010	0.13 ± 0.03	1.24 ± 0.10	1.16 ± 0.06	1.05 ± 0.03

Listed are mean values ± SD from 3 to 4 independent experiments

## Discussion

### Proton RBE

We report the RBE values of the first therapeutic 60-MeV proton beam in Poland, operated at IFJ PAN. Radiobiological experiments, performed in vitro on primary normal human fibroblasts with cell survival as end point, show RBE values for this beam to fall in the range 0.95–1.24 depending on survival level, position within the non-modulated and modulated proton beam, and patient.

Most of the in vitro experiments with proton beams were performed on continuous, mainly tumour cell lines. In the present study, we used normal primary fibroblasts from patients with cervix cancer. Primary cells are certainly better model of the in vivo condition, but they may produce greater variability between individual experiments. That is why, despite the same restrictive conditions applied in our study, the mean *α*-values for reference radiation are different for fibroblasts of the same patient (e.g. for HFIB15 is 0.627 in Table 1 and 0.446 in Table 2). The experiments with the modulated Bragg peak (and X-rays) were performed a few months later than those with the pristine proton beam (and X-rays). Therefore, we decided not to combine the reference radiation data especially that this would not improve the determination of RBE values.

In the present study, the mean RBE for fibroblasts irradiated near the maximum of pristine Bragg peak was found to range between 1.06 and 1.15 at SF = 0.01 and to reach a value between 1.03 and 1.21 at SF = 0.37, depending on the patient (Table 1). Petrović et al. (2006), at 2 Gy and γ reference radiation, reported a much higher RBE value of 1.69 for a human HTB140 melanoma cell line irradiated close to the Brag peak maximum of a 62-MeV pristine proton beam. Because the LET_d_ value at the position near the pristine Bragg peak in ours (7.9 keV/µm) and Petrović’s (6.2 keV/µm) studies is comparable, such a large difference in RBE must be due to tissue type. Human skin fibroblasts used in our study are radiosensitive cells in which the SF2 value ranges between 0.21 and 0.28, while HTB140 melanoma cells are extremely radioresistant with a SF2 value of 0.95. It was shown by Belli et al. (2000) that RBE for high-LET protons (30 keV/µm) depends on cell radiosensitivity to γ-rays, being the highest (3.19) for the most radioresistant cell line (SQ20B with SF2 = 0.72), and the lowest (0.82) for the radiosensitive (SF2 = 0.28) normal human lung fibroblasts. Moreover, these fibroblasts responded in a similar fashion to changes in LET_d_ from 7.7 to 29 keV/µm, attaining RBE value ranging merely from 0.86 to 0.82.

Most of our knowledge about the response of normal tissues to radiation has been obtained from studies utilising human skin as a model. To provide evidence to support the claim that RBE is dependent on radiosensitivity, ideally, proton RBE would be determined for two types of skin cells: fibroblasts and keratinocytes (at least twice as resistant as fibroblasts) (Słonina et al. 2007). Keratinocytes are target cells involved in acute epidermal reactions to radiotherapy, whereas fibroblasts are target cells responsible for late effects (fibrosis) in skin and other organs (i.e. lung, kidney, and bowel). Although we have both types of cells, derived from patients with cervix cancer (Słonina et al. 2007), the PE of keratinocytes was too low (~0.8–3 %) to get credible cell survival results in the present study.

Since a pristine Bragg peak is too narrow to cover the clinical target (tumour) volume, for radiotherapy, it has to be spread out (modulated). Therefore, those RBE values are clinically useful that are calculated for modulated proton beams used in radiotherapy. Because in proton therapy of uveal melanomas high doses per fraction (13.64 Gy) are applied, the RBE value evaluated at low survival level (0.01) related to our higher doses (~5 Gy in the case of human fibroblasts) is of clinical relevance. In our study, the mean RBE values measured at SF = 0.01 at three different depths within the SOBP ranged between 0.95–1.00 at 2 mm, 0.97–1.02 at 15 mm, and 1.05–1.11 at 27 mm (Table 2). Thus, the range of RBE values at the end of the SOBP (1.05–1.11) corresponds well with that close to the Bragg peak maximum in the pristine beam (1.06–1.15). We would not expect the clinically relevant RBE values at the dose of 13.64 Gy to be any different from those we assessed at SF = 0.01. These RBE values are very close to the values of RBE of 1.0 and 1.1, commonly accepted in clinical practice.

### Proton RBE as a function of dose

It is well known that the RBE after high-LET radiation (heavy ions, neutrons) increases with decreasing dose in vitro or decreasing dose per fraction in vivo (Joiner 2009). This is because survival curves for low-LET reference radiations have a shoulder (corresponding to higher cell survival) in the low-dose region. As late-responding tissues have a larger shoulder (lower *α*/*β* ratio), variation in RBE with dose is more pronounced than that in early-responding tissues with a higher *α*/*β* ratio (Gerweck and Kozin 1999). Although the biological effects of protons are considered to be similar to those of photons, any significant increase in RBE with decreasing irradiation dose (or increasing survival level), seen in some in vitro studies (Wouters et al. 1996; Tang et al. 1997), may be explained by the clinical proton beams having a small high-LET component, especially at the distal part of the SOBP, as shown in Fig. 2.

In our study, the RBE values gradually increased with survival level, from 0.01 to 0.37 (corresponding to the dose level decreasing from ~5 to ~1 Gy) at the position near the pristine Bragg peak maximum (Table 1), as well as at any depth of the SOBP (Table 2). This increase was not significant, except for the case of HFIB30 fibroblasts. Here, at the distal part (27 mm) of the SOBP (position at which the effect could be expected), the RBE increased significantly from 1.05 at SF = 0.01 to 1.24 at SF = 0.37, which we take to be an individual case. Although skin fibroblasts, as target cells of a late-responding (slowly proliferating) tissue with a low *α*/*β* ratio, might be expected to show variation in RBE with dose, as radiosensitive cells in vitro exhibit survival curves with a small shoulder (Figs. 3, 4) and quite high *α*/*β* ratio (Tables 1, 2) and weak RBE–dose dependence.

In the present study, the RBE values for normal human fibroblasts measured at SF = 0.01 (~5 Gy) within the SOBP were low and at 2 and 15 mm depths, the values were equal or slightly below 1.0, while at depth of 27 mm it ranged from 1.05 to 1.11 (Table 2). These experimental results seem to be consistent with the theoretical analysis recently published by Carabe et al. (2012) and Dasu and Toma-Dasu (2013). The authors studied the dependence of the RBE and biologically effective range of the proton beam, on the LET, tissue type (its *α*/*β* value) and dose per fraction and found that the RBE is below 1.1 for large doses and large values of *α*/*β*, which translates into a shorter biological range than this obtained with an RBE of 1.1 (Carabe et al. 2012).

Blomquist et al. (1993) using 67-MeV protons found an RBE–dose dependence (RBE 1.15 at SF = 0.01 increased to 1.63 at SF = 0.5) only for radioresistant V79-379A hamster cells (characterised by survival curves with a shoulder) but not for radiosensitive human colon carcinoma LS174T cells (described by survival curves without such a shoulder). On the other hand, Petrovic et al. (2006), irradiating very radioresistant human melanoma HTB140 cells with a 62-MeV proton beam, did not find any change in RBE as the dose decreased from 16 to 2 Gy. For human cells, no significant RBE variation with survival levels was found by Ando et al. (2001) who used 235 MeV protons and normal fibroblasts (NB1RGB) or head and neck carcinoma SCC61 cells, nor was any RBE variation seen by Kagawa et al. (2002) in salivary gland cancer (HSG) cells exposed to a 190-MeV proton beam. For human skin fibroblasts irradiated at the mid-range of the SOBP, Ando et al. (2001) found the same RBE value of 1.1 at SF2 and at SF = 0.1. Kagawa et al. (2002) reported the same range of RBE values within the SOBP at SF = 0.5 (1.01–1.07) and at SF = 0.1 (1.01–1.05). Our RBE values for our 60-MeV SOBP calculated at SF = 0.1 (0.99–1.16) are close to those discussed above.

Similarly, several in vitro Chinese hamster cell survival studies have not indicated any change in RBE as the dose decreased, such as the studies of Yashkin et al. (1995) who irradiated clone 431 cells with 179 MeV protons, or of Gueulette et al. (1996) in CHO cells exposed to 85 MeV protons. Over the centre of the SOBP, Yashkin et al. (1995) obtained RBE values of 1.07 and 1.08 at SF = 0.1 and SF = 0.37, respectively. However, for the same cell lines, contradictory results were also reported. Wouters et al. (1996) using a 70-MeV proton beam demonstrated a significant effect of irradiation dose on RBE throughout the SOBP at doses below 4 Gy. In their study, the RBE value for V79 cells increased from 1.37 at 2 Gy to 1.56 at 1 Gy. Tang et al. (1997) applying 65 MeV protons to CHO cells showed significant increase in RBE as the dose decreased from about 8 to 1 Gy at each studied depth of modulated proton beam. As mentioned earlier, in our 60-MeV proton beam used for the therapy of uveal melanomas, the dose dependence of RBE is of no concern, due to the high dose per fraction (13.64 Gy) delivered in this treatment.

### Proton RBE as a function of depth in the SOBP

The SOBP is composed out of many pristine Bragg peaks of varying intensity and range which are superimposed. In consequence, a gradual increase in LET can be observed within proximal to distal parts of the SOBP (Courdi et al. 1994; Wouters et al. 1996). According to Grassberger et al. (2011), LET value of 5–15 keV/µm can be expected at the distal part of clinically used proton beams. In the present study, for fibroblasts of each patient, the RBE value increased at the distal part (27 mm) of the 60-MeV SOBP (Table 2), although the RBE–depth dependence was significant for fibroblasts of one patient only, i.e. HFIB30. Here, the RBE value at depth of 27 mm was significantly higher than that at 2 mm of the SOBP at all three survival levels. This is consistent with the depth–LET_d_ distribution within the SOBP, as shown in Fig. 2. The SOBP LET_d_ value at the depth of 2 mm (2.25 keV/µm) rises to 7.50 keV/µm at 27 mm depth. While the LET_d_ value at the depth of 15 mm (2.93 keV/µm) was comparable to that at 2 mm, the difference in RBE at 15 and 27 mm depths of the SOBP was not significant.

The variation in RBE at different initial proton energies and different depths in the SOBP has been investigated by many authors. The RBE–depth relationship has been reported mostly for proton beams of intermediate energies, 60–87 MeV. In the studies by Bettega et al. (2000) performed on human tumour SCC25 cells irradiated with the 65 MeV protons, the RBE values (at SF = 0.1) increased with increasing depth from 1.04 at the proximal to 1.22 at the distal part of the SOBP. Courdi et al. (1994) evaluated the RBE at four positions in the 65-MeV half-modulated SOBP. For human melanoma cells, the RBE values at SF = 0.01 were 1.09, 1.12, 1.19, and 1.27 at 15, 20, 25, and 26.8 mm depths of the SOBP, respectively. Calugaru et al. (2011) confirmed the variation in the RBE with depth in their 76-MeV SOBP but not in the 201-MeV SOBP. The RBE values for two human tumour cell lines varied between 1.07 and 1.09 at the mid-SOBP and grew to 1.25 and 1.22 at the distal part of the 76-MeV SOBP. In contrast, Britten et al. (2013) recently found much higher RBE values along the 87-MeV SOBP for human Hep2 laryngeal cancer cells. Their RBE value was 1.46 at the centre of the SOBP and increased to 2.1 at its distal end. The values correlated with the LET_d_ values at these depths of 5.3 and 20.5 keV/µm, respectively. The latter LET_d_ value is much higher than ours (7.9 keV/µm) and that expected at the distal part of the SOBP of a proton beam used clinically (Grassberger et al. 2011). An increase in the RBE with depth, though not significant, was also demonstrated by Blomquist et al. (1993) for human LS174T cells irradiated with 67 MeV protons and by Gueulette et al. (1996) who obtained RBE values of 1.01 at the entry and 1.09 at the distal end of a 3-cm SOBP (at SF = 0.01). Significant increase in biological effectiveness with depth in the SOBP was also demonstrated for Chinese hamster cells (Tang et al. 1997; Wouters et al. 1996; Britten et al. 2013) in 65- to 87-MeV proton beams. The authors stated RBE values ranging between 1.2 and 1.5 at the distal part of the SOBP. Although the magnitude of this RBE variation seems to be rather low, in hypofractionated radiotherapy (such as applied in treating uveal melanoma), critical organs located just behind the target volume (at the end of the SOBP) could receive a physical dose that is biologically more effective. For this reason, the clinical relevance of the RBE variation with treatment parameters and the range uncertainty of the proton beam need to be extensively studied.

In overall conclusion, the RBE values for normal human fibroblasts estimated at SF = 0.01, which we have established for the 60-MeV proton beam at IFJ PAN in Krakow, Poland, are close to the values of RBE of 1.0 and 1.1, routinely applied in clinical practice.
